# Mid-infrared wearable biosensing for metabolic syndrome: Opportunities and translational challenges

**DOI:** 10.1016/j.isci.2026.116633

**Published:** 2026-07-02

**Authors:** Lampros Androutsos, Thomas Birngruber, Ivana Campia, Natalia Escacena, Alexandru Floares, Carmen Floares, Nikolaus Hahne, Jan Kischkat, Maaz Mohsin, Sheraz Naseer, Sabine Oertelt-Prigione, Thomas Pieber, Heikki Saari, Raphael Schlesinger, Maxine Silvestrov, Negin Soroush, Marco Straccia, Oliver Supplie, Hermann Von Lilienfeld-Toal, Oili M.E. Ylivaara, Adrian Zety

**Affiliations:** 1 Artificial Intelligence Expert SRL, Cluj Napoca, Romania; 2 Joanneum Research Forschungsgesellschaft mbH HEALTH - Institute for Biomedical Research and Technologies, Graz, Austria; 3 FRESCI by Science&Strategy SL, Barcelona, Spain; 4 Quantune Technologies GmbH, Berlin, Germany; 5 Gender Unit, Department of Primary and Community Care, Radboud University Medical Center, Nijmegen, the Netherlands; 6 VTT Technical Research Centre of Finland, P.O. Box 1000, Espoo 02044, Finland

**Keywords:** mid-infrared spectroscopy, wearable biosensors, interstitial fluid monitoring, metabolic syndrome, digital health

## Abstract

Metabolic syndrome (MetS) is a multifactorial condition associated with an increased risk of type 2 diabetes, cardiovascular disease, and other comorbidities. Current diagnosis relies on episodic clinical assessments and invasive laboratory tests, limiting early detection and continuous monitoring in real-world settings. Digital health technologies, particularly wearable biosensors, may help address these limitations through continuous and user-centered monitoring. The MiWear project explores miniaturized mid-infrared (Mid-IR) spectroscopy combined with artificial intelligence (AI) to support non-invasive biomarker monitoring in interstitial fluid (ISF). By focusing on Mid-IR molecular signatures relevant to MetS, the approach could enable earlier risk stratification and longitudinal metabolic assessment outside traditional clinical environments. We discuss sex- and gender-related considerations, analytical and clinical validation needs, regulatory pathways, and implementation scenarios across primary care, population health, and telemedicine. This translational roadmap highlights opportunities and challenges for integrating Mid-IR wearable biosensing in preventive, patient-centered metabolic healthcare.

## Teaser

biodevices; medical device in health technology; metabolic flux analysis

## Background

Metabolic syndrome (MetS) is a multifactorial condition characterized by the clustering of interrelated metabolic abnormalities, such as abdominal obesity, hypertension, impaired fasting glucose, elevated triglyceride levels, and reduced high-density lipoprotein cholesterol (HDL-C), that collectively increase the risk of developing type 2 diabetes (T2DM) and cardiovascular diseases^1^^,^^2^ while also reflecting a systemic disorder with multisystem involvement extending to non-alcoholic fatty liver disease (NAFLD) and other malignancies, including certain cancers.^3^^,^^4^ Excess visceral fat is a key driver of these alterations by acting through dysregulated adipokine secretion and neurohormonal activation. Genetic predisposition, circadian disruption, and environmental factors such as diet and physical inactivity further contribute to its development.^5^ Chronic low-grade inflammation is also recognized as a pivotal mechanism linking central adiposity to insulin resistance and cardiometabolic risk,^6^ while gut microbiota may further modulate metabolic regulation.^7^ Although not formally classified as a disease, MetS is increasingly recognized as a major public health concern, imposing substantial socioeconomic pressure on healthcare systems worldwide.^8^ MetS is associated with increased cardiovascular and all-cause mortality, with prevalence varying significantly by age, sex, and geographic region.^9^^,^^10^^,^^11^^,^^12^^,^^13^ Several studies report global prevalence ranging from ∼12.5% to 31.4%, depending on diagnostic criteria,^11^ with prevalence increasing markedly with age^12^ and influenced by sex- and gender-related factors,^14^ MetS is increasingly being observed in younger populations due to rising obesity rates,^15^ with prevalence ranging from 0.3% to 26.4% in children and adolescents, depending on diagnostic criteria.^16^^,^^17^

Lifestyle interventions remain the cornerstone of MetS management,^18^ while wearable and smartphone-based systems are increasingly being explored to support scalable prevention and continuous metabolic monitoring, despite persistent challenges in adherence, standardization, and real-world implementation.^19^^,^^20^^,^^21^ In this context, emerging wearable sensing platforms integrating advanced spectroscopic techniques and AI-driven data analysis, including mid-infrared (Mid-IR) approaches, represent a promising direction for the non-invasive metabolic assessment.

## Scientific and clinical gaps

Despite advances in understanding MetS pathophysiology, current clinical approaches remain largely episodic and reactive, highlighting the need for continuous monitoring strategies capable of capturing early metabolic alterations.

### Diagnostic limitations and disconnect between biomarker knowledge and use

Current diagnostic approaches for MetS rely on periodic clinical assessments based on surrogate clinical markers, including waist circumference, triglycerides, HDL-C, blood pressure, and fasting blood glucose.^19^^,^^22^ These assessments are inherently episodic and rely on discrete clinical time points, which may fail to detect subtle metabolic changes or transient physiological fluctuations, delaying early identification of affected patients. While routine biomarkers, such as fasting glucose and triglycerides, are widely used, there remains a lack of accessible, non-invasive approaches to monitor key metabolic parameters in real time, including aspects of insulin resistance, lipid metabolism, or inflammation.^23^ In this context, uric acid and ketone bodies are increasingly being investigated as indicators of early metabolic dysregulation, and lactate has been reported as a marker of impaired oxidative metabolism and insulin resistance.^24^ Serum uric acid level has been associated with MetS and proposed as a potential early risk biomarker beyond conventional diagnostic criteria. In a prospective cohort, uric acid was associated with incident MetS, supporting its potential role in risk stratification.^25^ Wearable platforms have demonstrated the feasibility of non-invasive uric acid monitoring in biological fluids. Consistently, uric acid has been validated as a salivary biomarker correlated with MetS and integrated into emerging wearable platforms for cardiometabolic risk monitoring.^26^^,^^27^ In parallel, glucose and ketone dynamics are increasingly being incorporated into metabolic risk models to improve stratification beyond standard clinical criteria.^28^ Several robust biomarkers, including inflammatory cytokines, oxidative stress markers, uric acid, ketone bodies, and lipid-related components, have been identified and increasingly being explored through wearable biosensing approaches,^28^^,^^29^ yet, their clinical implementation remains limited by challenges in standardization and cross-population validation. Recent advances in wearable biosensing technologies have demonstrated the feasibility of continuous monitoring of such metabolites in interstitial fluid (ISF), including real-time β-hydroxybutyrate (BHB) tracking through continuous ketone monitoring systems as well as microneedle-based wearable patches enabling dynamic *in vivo* metabolic monitoring.^30^^,^^31^ Continuous monitoring of these biomarkers could enable earlier detection of metabolic deterioration and support personalized interventions, bridging the gap between research discovery and actionable patient care.^32^^,^^33^ However, their clinical implementation remains limited by challenges in standardization and cross-population validation. This translational gap highlights the need for continuous and scalable monitoring approaches capable of integrating biomarker discovery into real-world metabolic health assessment. There is a clear need for continuous, real-time monitoring technologies that can support earlier identification and intervention, particularly to enable the detection of metabolic deterioration during early (prodromal) stages, before the onset of overt clinical symptoms, as suggested by emerging translational initiatives such as the EIC-funded MiWear project.^34^

### The impact of sex differences on metabolic syndrome

Sex-specific criteria have been integral to defining MetS since the WHO’s original framework,^35^ which distinguished thresholds for HDL-C and central abdominal obesity in male and female patients. Subsequent definitions by major health organizations, including the International Diabetes Federation (IDF) and the National Cholesterol Education Program Adult Treatment Panel III (NCEP-ATP III),^36^ have consistently maintained these sex-specific cut-offs for HDL-C and waist circumference, with some also introducing ethnicity-based adjustments. Across definitions, sex remains a key variable in evaluating MetS risk factors. A growing body of evidence indicates that men and women exhibit distinct patterns in both the onset and clinical manifestation of MetS. Prevalence rises more steeply with age in women than in men.^37^ Men tend to develop MetS earlier, often presenting with elevated triglyceride levels and higher blood pressure, whereas women are more likely to experience increased abdominal adiposity, insulin resistance, and pro-inflammatory biomarker profiles, particularly after menopause.^37^^,^^38^^,^^39^ Body fat distribution patterns differ markedly between sexes, with men tending to accumulate more visceral fat, while women typically present higher subcutaneous fat mass distribution.^40^ Both adipose depots are metabolically active, acting as endocrine organs that secrete adipokines and other regulatory factors,^41^ but visceral adipose tissue (VAT) exhibits a more pro-inflammatory profile and a stronger association with cardiometabolic risk.^42^ Consequently, differences in fat distribution contribute to sex-specific metabolic risk profiles and disease trajectories. A higher proportion of VAT represents a significant risk factor for MetS and mortality in older individuals of both sexes, even among individuals with normal body weight,^43^ and is also associated with cortisol production and metabolism dysregulations.^41^ Hormonal regulation further shapes sex-specific risk, as estrogens exert protective effects against metabolic dysfunction in premenopausal women, whereas postmenopausal estrogen decline contributes to increased visceral adiposity and clustering of metabolic risk factors central to MetS.^37^ Beyond biological factors, sex and gender also influence engagement with health technologies, affecting adoption, adherence, and data representativeness in wearable systems. Combining considerations such as biological characteristics with gender-related behavioral and social factors, including caregiving roles and differential access to technology, into wearable technology design is essential to address health inequalities that disproportionately impact women and marginalized populations. Inclusive design and validation strategies are, therefore, required to improve accessibility, acceptability, and performance across populations.^44^^,^^45^ However, current digital health tools often fail to adequately integrate gender and socio-economic factors, risking reinforcement of existing health inequalities.^46^ Despite increasing awareness, gender-related aspects remain widely underrepresented in human-centered design, even though they influence health perception, digital literacy, care-seeking behavior, and engagement with digital health technologies.^47^^,^^48^ Addressing these dimensions is essential for equitable and reliable deployment of wearable metabolic monitoring technologies.

### Skin considerations for wearable biosensors

Skin structure characteristics, which vary by sex, age, and hormonal status, are highly relevant in the context of wearable biosensors. Sex-related biological differences can affect epidermal thickness, hydration, sweating levels, and hair growth, which may influence sensor performance and signal reliability.^49^ These differences are not only structural but also functional, as variations in sweat rate, sweat composition, and skin pH between men and women can directly affect the accuracy and stability of electrochemical and optical measurements in wearable devices.^50^ Furthermore, individual differences in skin elasticity, compliance, and microcirculation may modulate sensor-skin contact and signal quality, thus highlighting the need for adaptive and flexible sensor design. These factors highlight biological heterogeneity as a key constraint for transcutaneous sensing rather than a secondary source of noise. Emerging flexible and stretchable skin-like sensors offer mechanical compatibility with human skin, and they allow conformal attachment and maintain signal fidelity despite motion or morphological differences.^51^ These devices are capable of detecting a wide range of physiological signals, including thermoelectrical, neural electrical, photoelectrical, electrochemical, and mechanical pressure signals, and enable continuous, long-term, non-invasive health monitoring.^52^ However, despite these advances, physiological variability across populations remains a key challenge for robust signal interpretation, particularly in real-world conditions where motion artifacts, environmental changes, and inter-individual skin differences coexist. Evidence from wearable systems demonstrates that tailoring sensor design and calibration to individual physiological characteristics improves comfort and data quality, supporting the need for population-specific validation and standardization protocols.^53^ Accounting for these factors during validation studies can enhance device performance, increase user adherence, and support more equitable application across diverse populations.

## Wearable metabolic monitoring: Opportunities and translational challenges

Despite significant advances in wearable sensing technologies and biomarker discovery, the translation of these developments into clinically robust, continuous monitoring systems remains an ongoing challenge. Current approaches are often based on enabling technologies that demonstrate feasibility at laboratory or proof-of-concept level, but they still face limitations in integration, long-term stability, and clinical validation in real-world conditions. ISF has emerged as a key target biofluid for minimally invasive and continuous monitoring due to its biochemical similarity to blood and accessibility via skin-interfaced devices.^54^ However, most systems remain constrained by indirect sensing strategies, limited clinical validation, and insufficient integration into real-world healthcare workflows. The integration of high-throughput biomarker discovery with wearable quantification strategies has been proposed as a promising direction, yet it remains largely at an early translational stage.^55^ In this context, optical sensing approaches such as Mid-IR spectroscopy have demonstrated strong potential for label-free biochemical detection, but they also reveal fundamental limitations in transdermal measurement due to scattering, absorption by water, and depth-related constraints.^56^ Recent studies suggest that improved spatial localization of the optical signal within skin layers can partially mitigate these effects and enhance detection in blood-rich compartments,^57^ although clinical *in vivo* applications remain limited. These limitations are fundamentally associated with strong wavelength-dependent absorption in biological tissues, particularly water, proteins, and lipids, which leads to shallow effective penetration depths and rapid signal decay within superficial skin layers,^53^^,^^58^^,^^59^ with reported attenuation lengths in the order of a few to tens of micrometers depending on wavelength and tissue composition.^56^^,^^60^ This restricts direct optical access to ISF biomarkers and often necessitates indirect or surface-coupled measurement strategies. In recent translational studies, Mid-IR glucose sensing systems have reported mean absolute relative differences of ∼20% in early-stage clinical evaluations,^61^ while minimally invasive electrochemical microneedle-based platforms have demonstrated high analytical sensitivity and rapid response times,^62^ highlighting the diverse performance-invasiveness trade-offs across current sensing paradigms. Overall, these findings suggest that the field has progressed from feasibility demonstrations toward early translational validation but remains limited by fundamental issues in signal specificity, biological variability, and real-world robustness. Together, current wearable metabolic monitoring approaches remain fragmented, underscoring the need for integrated, clinically validated systems.

## MiWear project: An innovative breakthrough approach

MiWear can be positioned within the emerging landscape of integrated wearable metabolic monitoring approaches as a European collaborative effort aimed at advancing continuous, non-invasive assessment of metabolic biomarkers for early detection and management of MetS. MiWear^34^ is a three-year project funded under the European Innovation Council Pathfinder challenge “Towards the Healthcare Continuum: Technologies to support a radical shift from episodic to continuous healthcare”.^63^ The project involves a multidisciplinary European consortium from small-medium enterprises (SMEs), research institutions, and academia, with expertise spanning biomarkers, optical spectroscopy, laser physics, artificial intelligence (AI), and translational healthcare domains, including business management, financial strategy, science communication, and the gender-specific dimensions of biomarker research. The framework builds on recent advances in integrated sensing strategies for the measurement of selected biomarkers relevant for MetS and explores the potential of miniaturized Mid-IR spectrometry, traditionally limited to laboratory settings. Target biomarkers include glucose, BHB, lactate, and uric acid, selected based on their relevance for metabolic dynamics and potential for continuous monitoring, providing early detection from pre-symptomatic stages. Mid-IR spectroscopy enables label-free interrogation of molecular vibrational signatures in biological tissues, providing access to metabolically relevant information in ISF and supporting non-invasive biomarker quantification. Within this framework, MiWear investigates the integration of Mid-IR sensing into wearable-compatible architectures, with ongoing validation progressing from analytical and biological matrix evaluation toward subsequent *ex vivo* and *in vivo* clinical validation stages and supporting the objectives of the EIC healthcare continuum challenge. MiWear builds upon prior successful projects carried out by consortium partners, which have:

1. Established correlations between specific biomarkers and MetS;
2. Characterized biomarker absorption spectra within the Mid-IR range (8–11 μm); and
3. Validated the feasibility of using Mid-IR spectrometers at higher wavelengths for measuring glucose biomarkers.

The project establishes a strategic roadmap for the systematic development and translational pathway of MiWear technologies. Beginning with the consolidation of the technology concept, MiWear progresses toward experimental proof of concept, laying the foundation for subsequent technological advancements and future deployment. MiWear activities encompass:

1. The technological development of miniaturized Mid-IR spectrometers;
2. Investigation of MetS biological aspects and key biomarkers detectable in midIR spectrum; and
3. Clinical, regulatory, and business assessments to support future deployment and market translation.

**Figure 1:**
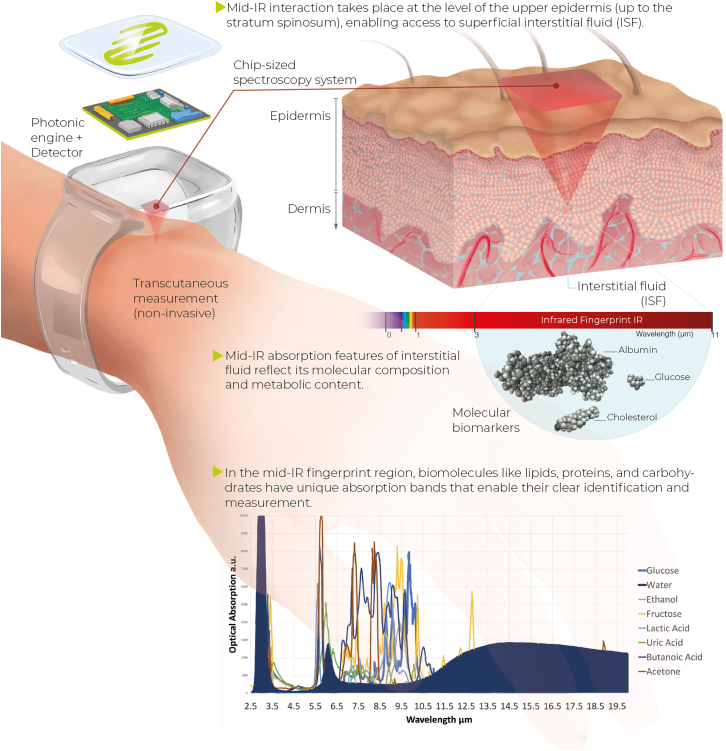
MiWear technological development workflow, from biomarker identification and artificial intelligence integration to prototype validation and regulatory assessment The project combines multi-disciplinary expertise to advance Mid-IR sensing from concept to clinical translation. Original figure created for this manuscript; illustrations by Dasha C. del Blanco.

Continuous biomarker tracking within a structured validation framework represents a concrete step toward more proactive and preventive metabolic health management. An overview of the project strategy is presented in Figure 1, while the following sections explore its technological, clinical, and societal dimensions in greater depth.

## Technology innovation: Mid-infrared spectroscopy

**Figure 2:**
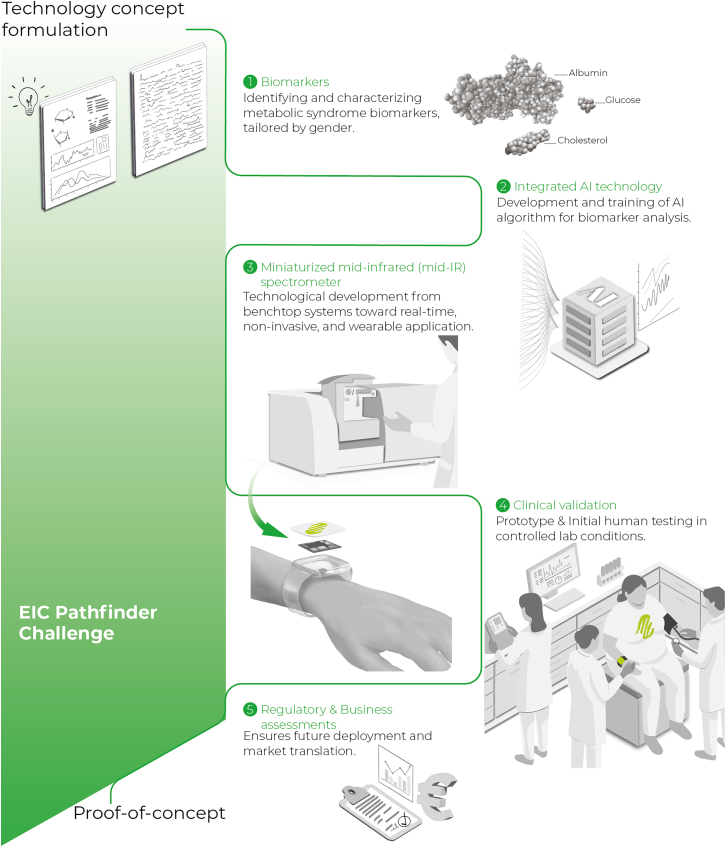
Principle of transcutaneous mid-infrared spectroscopy used in MiWear The wearable device emits mid-infrared (Mid-IR) light through the skin to capture molecular vibration signals from interstitial fluid, enabling non-invasive analysis of metabolic biomarkers. Artificial intelligence algorithms interpret these signals to provide continuous, real-time metabolic monitoring. Original figure created for this manuscript; illustrations by Dasha C. del Blanco.

The MiWear project is focused on the development and validation of an innovative Mid-IR spectroscopic platform tailored for wearable applications (Figure 2). This section outlines its technological foundations, describing the physical principles of Mid-IR spectroscopy and its diagnostic potential for non-invasive biomarker detection.

### Principles and diagnostic potential of mid-infrared spectroscopy

Mid- IR spectroscopy operates in the 2.5–25 μm (4,000–400 cm^−1^) region of the electromagnetic spectrum, commonly referred to as the molecular fingerprint region. Within this range, virtually all biomolecules exhibit fundamental vibrational modes corresponding either to specific chemical bonds or the entire chemical/molecular ensemble, thus producing highly specific sharp bands, generating characteristic spectral signatures for biomolecules such as lipids, proteins, and carbohydrates.^64^^,^^65^ This property enables, in principle, the differentiation and quantification of multiple biomolecular species, although performance remains dependent on signal quality and tissue-related constraints. The present project applies mid-IR spectroscopy to measure selected biomarkers, relevant to metabolic processes underlying MetS. Characteristic spectral regions associated with proteins, lipids, and carbohydrates have been correlated to altered metabolic conditions.^66^ By probing tissues or biofluids, Mid-IR light can capture molecular fingerprints of key metabolites, enabling the detection of physiological and pathological changes linked to metabolic imbalance.

Mid-IR spectroscopy has demonstrated strong performance in monitoring glucose, lipids, proteins, and other clinically relevant analytes, with early applications explored in metabolic disease contexts.^67^^,^^68^ Proof-of-concept systems suggest detection limits in the low mg/dL range for selected analytes, with target sensitivity in the order of <10 mg/dL and temporal resolution in the range of a few minutes for continuous monitoring applications, although sensitivity, temporal resolution, and calibration stability remain strongly system-dependent.^69^^,^^70^^,^^71^ Recent technological advances within the MiWear project focus on translating Mid-IR spectroscopy from benchtop setups to miniaturized, skin-contact wearable devices. Key enabling innovations include: compact, tunable quantum cascade lasers (QCLs), photonic integrated circuits (PICs), computational spectrometers, micro-electro-mechanical systems (MEMS)-based tunable filters, and machine learning (ML) algorithms for spectral reconstruction and calibration.^72^^,^^73^ Together, these developments support the design of integrated optical-computational platforms for wearable metabolic sensing. In this context, MiWear explores the integration of these components into a wearable-compatible architecture for ISF sensing.^34^ Analysis of backscattered light or attenuated spectra allows detection of metabolites such as uric acid, glucose, ketone bodies, and albumin.^68^^,^^71^^,^^74^ The diagnostic potential of Mid-IR spectroscopy lies in its ability to: (1) provide label-free, reagent-free biochemical quantification; (2) offer molecular-level specificity across multiple biomarkers simultaneously; and (3) enable continuous and personalized metabolic profiling when integrated into wearable platforms.^68^

### Positioning Mid-IR among optical techniques for metabolic biomarker detection

Optical spectroscopy modalities such as near-infrared (NIR), Raman, and fluorescence-based sensing have all been explored for metabolic biomarker detection, but each presents limitations in specificity, robustness, or translational readiness. For example, NIR spectroscopy offers fast, low-cost screening, but its overtone and combination bands often overlap and therefore, reduce chemical discrimination and sensitivity.^75^ Raman spectroscopy provides high chemical specificity but suffers from weak scattering efficiency, strong fluorescence background, and limited penetration depth in biological tissues.^76^ Mid-IR spectroscopy similarly faces limited penetration depth, but provides access to fundamental vibrational modes of biomolecules, enabling label-free spectral information for key biomolecular classes. Beyond optical spectroscopy, other non-invasive and minimally invasive approaches include electrochemical sweat sensors, microneedle-based ISF sampling, and emerging implantable continuous biosensing platforms, each with distinct trade-offs in invasiveness, temporal resolution, and biochemical specificity.^54^^,^^77^ Recent advances in QCLs, MEMS Fabry-Pérot interferometers (FPIs), and computational reconstruction reduce size and power requirements, positioning Mid-IR as a promising platform for MetS screening and continuous metabolic monitoring.^71^^,^^78^ These advances collectively strengthen the potential of Mid-IR spectroscopy as a candidate modality for metabolic monitoring applications, with its potential balanced by ongoing challenges in system integration, signal robustness, and real-world validation. An overview of key metabolic biomarkers, along with their sensing approaches and translational maturity, is summarized in Table 1.

**Table 1:** Key metabolic biomarkers associated with MetS and corresponding wearable sensing strategies for non-invasive or minimally invasive monitoring

Biomarker	MetS relevance	Biofluid accessibility	Wearable/sensing approach	Translational status
Glucose	core marker of insulin resistance	blood and ISF	CGM, electrochemical sensors, and optical spectroscopy (NIR/Mid-IR)	clinically established continuous monitoring (diabetes); episodic use in MetS^22^^,^^28^^,^^79^
Ketone bodies (BHB)	lipid metabolism shift/metabolic stress	blood and ISF	electrochemical wearable ketone sensors	emerging continuous monitoring^30^^,^^31^
Uric acid	oxidative stress/insulin resistance/cardiometabolic risk	blood, saliva, and ISF	optical/electrochemical wearable sensing	epidemiologically associated with MetS risk^25^^,^^26^^,^^27^
Lactate	impaired oxidative metabolism/insulin resistance	blood and ISF	electrochemical sensing; emerging wearable approaches	associated with metabolic dysfunction; limited wearable integration^24^
Albumin	vascular dysfunction/metabolic progression	blood, urine, and ISF	microneedle-based ISF sensing; optical approaches	associated with MetS incidence; limited wearable validation^32^^,^^80^
Inflammatory cytokines	systemic inflammation	blood and ISF	emerging immunosensing platforms	high relevance; limited real-time wearable implementation^28^^,^^29^

Overview of key metabolic biomarkers in MetS, including physiological role, biofluid accessibility, wearable sensing modalities, and translational status ranging from established clinical use to early-stage wearable development. Technologies include CGM, Mid-IR/NIR spectroscopy, electrochemical sensors, and microneedle-based ISF platforms. ISF, interstitial fluid; CGM, continuous glucose monitoring; BHB, β-hydroxybutyrate; NIR, near-infrared.

### Miniaturization and integration in wearable devices

Conventional Mid-IR spectrometers are typically bulky, power-intensive, and confined to laboratory settings. In recent years, research advances in photonic integration, MEMS, and compact optics have driven exploratory efforts toward miniaturized Mid-IR systems for potential point-of-care applications; however, fully wearable implementations remain at an early developmental stage. Key technological drivers include QCLs, PICs, and computational spectrometers. QCLs are compact, tunable, and high-brightness Mid-IR light sources with wavelength-selective emission, which makes them suitable candidates for integration into chip-level spectrometer platforms for biochemical and biomedical applications.^72^^,^^73^^,^^81^ PICs enable on-chip routing and delivery of IR signals via integrated waveguides and micro-optical couplers, thus effectively replacing bulky mirrors and lenses while reducing device footprint and preserving optical performance.^82^^,^^83^ Computational spectrometers further miniaturize hardware by shifting spectral reconstruction to software algorithms and achieving high fidelity. MEMS-based tunable filters, such as FPIs, provide dynamic wavelength selection and compact spectral encoding, improving flexibility and analytical accuracy.^84^ Recent system-level approaches combine miniaturized Mid-IR components with computational signal processing and data-driven calibration strategies, although full integration into robust wearable systems remains an ongoing engineering challenge. These developments support emerging optical-computational architectures that couple hardware miniaturization with AI-assisted spectral reconstruction, representing a step toward wearable-compatible metabolic sensing.^34^

### MEMS components and hardware challenges

MEMS technologies are widely used as enabling components for miniaturization and integration of Mid-IR spectroscopic systems. These micro-electromechanical systems enable dynamic wavelength tuning, compact optical paths, and rapid spectral acquisition in platforms compatible with skin-contact or handheld operation. MEMS-driven structures, including cascaded optical stages and low-inertia actuators, support fast spectral tuning while maintaining mechanical stability under dynamic operating conditions and mechanical robustness under motion and vibration, which are critical for on-body applications. FPIs allow precise spectral selection within millimeter-scale form factors, forming a key building block for compact Mid-IR spectroscopic architectures with potential applicability in wearable sensing platforms.^85^^,^^86^^,^^87^

### MEMS Fabry-Pérot interferometers and their path toward wearable devices

Fabry-Perot interferometers (FPIs) are tunable optical filters consisting of two parallel mirrors separated by an optical cavity, where the mirror thickness and cavity spacing determine the operational wavelength range. The mirrors typically form distributed Bragg reflectors (DBRs) through alternating high and low refractive index layers.^84^ MEMS FPIs provide precise spectral selection in millimeter-scale form factors, enabling compact and tunable spectral filtering in miniaturized Mid-IR systems. Recent implementations have achieved high spectral accuracy and 5 nm resolution, supporting their use in compact spectroscopic architectures.^34^^,^^86^^,^^88^^,^^89^ Early MEMS FPIs, developed at Valtion Teknillinen Tutkimuskeskus (VTT) in the late 1990s and early 2010s, evolved from Si/SiO_2_ DBRs to Si-air designs,^90^^,^^91^^,^^92^^,^^93^ improving optical transmission, spectral resolution, and operational range from the TIR to visible/NIR. For wearable integration, compact 4 × 4 mm Si-air FPI chips with near-weightless mirrors exhibit stable performance regardless of orientation, while their low-mass mirrors require minimal actuation force and relatively low voltages, supporting integration into low-power portable systems. The main challenge lies in integrating the MEMS FPI with light sources and detectors into a compact package, while preserving low power consumption, thermomechanical stability, and small wearable size that can be mounted onto a wristband. MEMS FPIs remain a core optical element defining the size, cost, and performance of wearable devices.^34^^,^^86^^,^^88^

### From hardware bottlenecks to next-generation wearable Mid-IR devices

Despite remarkable progress in optical miniaturization, several hardware limitations continue to constrain wearable Mid-IR platforms. Signal strength and sensitivity are constrained by the limited optical path lengths in microscale geometries, reducing photon-matter interaction. Material compatibility poses challenges beyond ∼4 μm, as conventional silicon substrates absorb strongly at these wavelengths; alternative solutions, such as metamaterial-clad or suspended waveguides, can overcome this but increase fabrication complexity.^94^^,^^95^ Power consumption and thermal management are also critical: continuous-wave QCLs and MEMS actuation must operate efficiently to preserve battery life, maintain user comfort, and ensure stable performance, as localized heating can affect device stability and signal fidelity.^96^ Environmental robustness is essential for on-body applications, requiring MEMS components to maintain alignment, calibration, and signal quality under motion, temperature, and humidity fluctuations. Emerging system-level approaches, including current MiWear initiatives, are exploring how these bottlenecks may be addressed through the integration of QCLs, PICs, computational spectrometers, and MEMS-based tunable filters FPIs, combined with advanced calibration and signal-processing strategies. In this context, integrated platforms under development aim to combine hardware miniaturization with adaptive calibration, spectral decomposition, and energy-efficient actuation schemes to improve robustness under real-world conditions. These efforts highlight the importance of co-design between photonic hardware and computational reconstruction pipelines, rather than isolated component optimization. Co-design of MEMS-photonics architectures and on-chip thermal control strategies is expected to accelerate the translation of wearable Mid-IR spectroscopy into reliable platforms, paving the way for real-time, non-invasive metabolic monitoring.

## Artificial intelligence and data interpretation

In the MiWear project, an intelligent system for quantifying biomarkers from spectral data is being developed in collaboration with the AI expert (AIE) partner, representing a core innovation for spectral biomarker quantification in MetS monitoring. This approach leverages a hybrid framework that combines data-driven and knowledge-driven models, rooted in quantum chemistry, biophysics, biochemistry, and metabolic dynamics, and is designed to consider relevant variables such as age and biological sex. AI-driven methodologies, including ML, deep learning,^97^^,^^98^^,^^99^^,^^100^ explainable AI (XAI),^101^ and genetic programming symbolic regression (GPSR),^102^ are essential for interpreting the complex spectral data.^103^ GPSR is a distinctive ML approach capable of deriving mathematical models directly from experimental data. The AI pipeline follows a staged architecture reflecting the progression from controlled laboratory conditions to complex biological matrices. In early-stage development, a cascaded classification-regression framework is applied to aqueous and simplified samples. In biologically relevant matrices (e.g., dermal ISF [dISF]), the pipeline shifts toward direct quantitative modeling of individual biomarkers from composite spectral signals, due to overlapping spectral contributions, strong protein background, and inter-individual variability. The MiWear intelligent system is designed to transform raw spectral data into quantitative biomarker outputs. Training relies on databases of biomarker spectra from aqueous solutions and spiked dermal dISF samples, which are generated using gold-standard Mid-IR laboratory equipment. Model refinement is further supported through *ex vivo* human skin samples, thus enhancing the system’s ability to discern relevant signals amid biological background noise, with careful consideration of sex- and gender-related variations in biomarker profiles.^104^ The computational framework incorporates biomarker-specific regression models and systematic spectral preprocessing (baseline correction, normalization, and noise filtering) to improve robustness under biological conditions. Model validation follows a multi-level strategy, including cross-validation designed to avoid data leakage between biological samples, statistical significance testing, and independent physical validation through controlled spiking experiments in biological matrices. These steps ensure that extracted signals reflect true physicochemical information rather than artifacts or spurious correlations. Interpretability is addressed through feature-importance analysis (e.g., wavelength relevance mapping), enabling verification that predictions are driven by spectrally meaningful regions consistent with known Mid-IR absorption bands. Inter-subject variability is mitigated through subject-specific calibration or baseline correction strategies. To ensure practical applicability, the intelligent system is planned for evaluation in *in vivo* human studies to assess robustness under dynamic physiological conditions, minimize false positives, and ensure alignment with regulatory standards for clinical adoption. The finalized AI framework is designed for secure cloud-based deployment with wearable integration and will comply with relevant data protection standards (e.g., General Data Protection Regulation [GDPR]) to support future clinical translation.

## From lab to healthcare: The strategic roadmap

Emerging health technologies can create meaningful impact only when they progress from scientific development to clinical relevance, ultimately reaching systemic adoption. This is particularly crucial for MetS, which requires scalable, continuous, and user-friendly monitoring solutions. The Mid-IR-based wearable device represents this translational pathway, where clinical utility depends on a structured roadmap integrating robust validation, regulatory and ethical compliance, and targeted implementation strategies.

### From proof of concept to clinical utility: An operational validation pathway

Building on the strategic roadmap outlined earlier, for a Mid-IR-based wearable biosensor to progress from prototype to clinical use, the device must demonstrate the ability to deliver reliable, reproducible, and clinically meaningful information. Achieving this requires addressing both technical performance and contextual relevance, through a phased validation strategy aligned with current EU guidance on medical device software,^105^ ensuring regulatory compliance while supporting translation from research outcomes into healthcare practice. The validation process unfolds through three complementary stages:

(1) Analytical validation establishes the biosensor’s capability to detect metabolic biomarkers through transcutaneous spectral acquisition, confirming accuracy, precision, and robustness under variable real-world conditions.

(2) Clinical performance evaluation assesses whether device outputs are clinically meaningful, benchmarking them against reference laboratory diagnostics and across diverse populations stratified by age, sex, BMI, and comorbidities.

(3) Contextual relevance and usability address integration of the device into healthcare workflows, including user adherence, clinician acceptance, and its impact on patient engagement and clinical outcomes.

Together, these stages define a continuous translational pathway that bridges scientific discovery with regulated, patient-centered applications. This framework ensures that innovation is not only validated but also scalable, equitable, and sustainable within healthcare systems. This structured roadmap contributes to bridging the gap between technological innovation and clinical implementation. By coupling analytical rigor with contextual validation, it supports pathways toward regulatory alignment, patient trust, and sustainable integration within digital health ecosystems. Additional details for each stage are provided in provided in Table 2.

**Table 2:** Overview of key validation parameters and objectives across roadmap phases

Phase	Validation focus	Key metrics	Outcome/deliverable
Analytical validation	spectral detection of biomarkers	LoD, accuracy, precision, and robustness	verified spectral-to-biomarker reliability
Clinical performance	diagnostic correlation	comparison to lab gold standards and subgroup analysis	demonstrated clinical relevance differentiating application levels: • consumer-grade: wellness tracking, low accuracy, and minimal oversight • research-grade: rich data, limited validation, and not used for medical decisions • clinical-grade: high accuracy, regulated, and intended for diagnostic or monitoring use.
Usability and contextual relevance	real-world usability and user interaction	user adherence, clinician acceptance, and workflow integration	validated usability and reliability in real-world conditions; proven user and system-level feasibility

LoD, limit of detection.

### Regulatory and ethical considerations

The translation of wearable Mid-IR biosensing into clinical practice requires alignment with regulatory frameworks, data governance standards, and ethical considerations. Within the EU context, such systems are expected to fall under the medical device regulation (MDR), particularly when used for diagnostic or monitoring purposes, including software components classified as medical device software. Key requirements include robust clinical evaluation and validation across diverse populations to ensure equitable performance. Data governance and privacy are central considerations, requiring secure handling of personal health data in compliance with the GDPR. In parallel, usability and accessibility remain essential to support safe and effective real-world deployment. An overview of regulatory, ethical, and implementation considerations is provided in Table 3. Collectively, these aspects define the conditions for clinical reliability, user trust, and sustainable integration into healthcare systems.

**Table 3:** Regulatory and ethical requirements for wearable Mid-IR biosensors

Domain	Key reference	Core requirement	Implementation focus
Regulatory classification	MDR 2017/745; Medical Device Coordination Group (MDCG) 2019-11	class IIa or IIb; software qualifies as Medical Device Software (MDSW)	notified body involvement; clinical evaluation report (CER); post-market clinical follow-up (PMPF)
Data governance and privacy	GDPR (EU 2016/679)	user consent, transparency, and secure data handling	encrypted storage and processing; clear user information
Equity in validation	MDCG 2025-4	inclusion of diverse populations (age, sex, and ethnicity)	representative biomarker datasets; subgroup analysis
Usability and accessibility	ISO 14971	risk management, human factors, and adaptation to health literacy and physical limitations	interface design; workflow integration; usability testing
Ethical and societal considerations	MDCG 2025-4^105^	Promote trust, minimize misuse, and ensure societal legitimacy	ongoing monitoring, stakeholder engagement, adherence to ethical standards

### Adoption scenarios: Primary care, population health, and telemedicine

**Figure 3:**
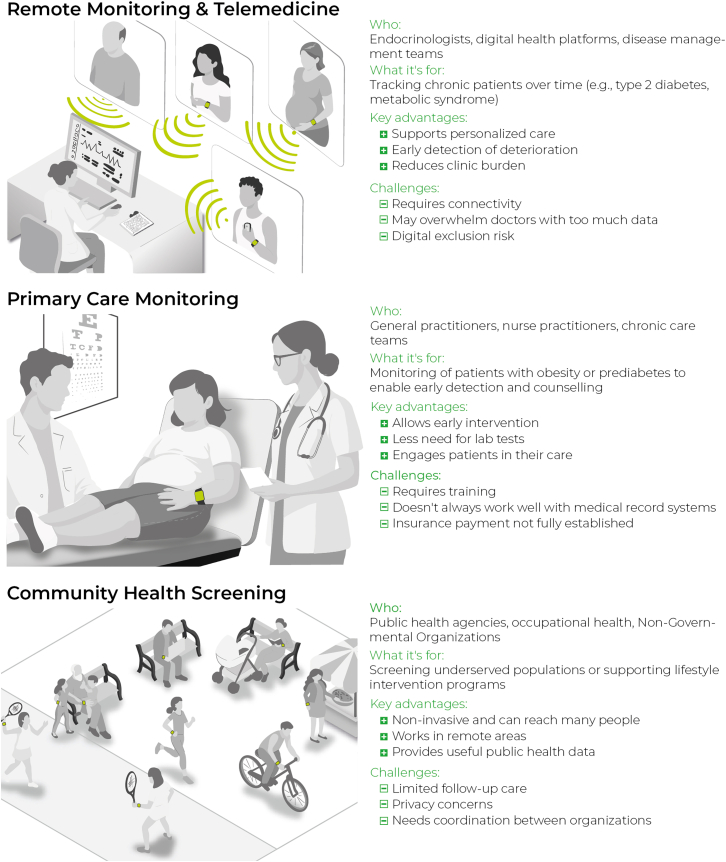
Strategic adoption scenarios for Mid-IR-based metabolic monitoring wearables The figure highlights key adopters, clinical applications, benefits, and limitations, illustrating actionable pathways for scalable integration across primary care, population health, and telemedicine. This framework underscores the potential of wearable metabolic monitoring to support early intervention, personalized care, and equitable healthcare delivery. Original figure created for this manuscript; illustrations by Dasha C. del Blanco.

The successful adoption of a Mid-IR-based wearable for metabolic monitoring depends on more than technical readiness and regulatory clearance. To achieve real-world impact, the technology must address concrete clinical needs across diverse healthcare settings, user populations, and system priorities. To bridge innovation with real-world impact, Figure 3 outlines three illustrative adoption scenarios—primary care, population health, and telemedicine—highlighting how such an approach may support precision prevention and broader access to metabolic monitoring. Each scenario maps stakeholders, use cases, and implementation challenges, emphasizing scalability and societal benefit. Key enabling factors include pilot deployments within real-world care pathways, user education for both patients and healthcare professionals, and design choices that promote equity, accessibility, and interoperability with existing digital health infrastructures. Together, these scenarios highlight how wearable Mid-IR spectroscopy could evolve from an enabling technology to a potential tool for early risk stratification and continuous metabolic health monitoring.

### Cross-cutting enablers across all scenarios

Beyond the specific contexts, several cross-cutting enablers are essential to ensure successful and sustainable adoption of Mid-IR-based wearables across healthcare systems.

1. Seamless integration with electronic health records (EHRs) and digital health platforms, ensuring efficient data flow and actionable decision support.
2. Clear and robust evidence of clinical utility and usability, fostering trust and confidence among clinicians and patients.
3. Alignment with digital health reimbursement frameworks, supporting economic sustainability and scalability.
4. A strong commitment to equity, accessibility, and trust, guaranteeing that technological benefits reach all populations inclusively and responsibly.

By strategically aligning technology, clinical workflows, and policy frameworks, Mid-IR-based wearables may contribute to improved metabolic health management, supporting early intervention, population-level insights, and more equitable healthcare delivery.

## Conclusion and call to action

The convergence of transdermal Mid-IR spectroscopy, device miniaturization, and AI defines a promising technological direction for preventive and personalized medicine. By embedding this technology in everyday wearables, it becomes possible to democratize access to molecular-level biochemical insights that are generally confined to laboratory testing, thus supporting shift from reactive treatment toward proactive and continuous metabolic health management. This transition directly addresses key limitations of current diagnostic pathways, which remain fragmented, episodic, and poorly suited to capturing early or dynamic metabolic changes characteristic of MetS. Importantly, continuous and non-invasive Mid-IR-based monitoring has the potential to reduce the long-standing disconnect between biomarker discovery and clinical use, translating rich biochemical knowledge into actionable, real-world diagnostics. When combined with AI-driven interpretation, wearable platforms can complement isolated risk factors toward integrated metabolic trajectories, supporting earlier and more personalized intervention strategies.

Realizing this potential requires coordinated and responsible action across research, policy, and healthcare systems.

• Rigorous and inclusive research and development (R&D), ensuring analytical accuracy, clinical validity, and robust AI models, with attention to sex- and gender-specific differences, dataset bias, and population diversity.
• Adaptive regulatory frameworks and equitable funding mechanisms, supporting high-impact digital innovation.
• Strategic implementation within healthcare systems, anchored in policies and reimbursement models that prioritize early detection over late-stage intervention, including integration into primary care, telemedicine, and remote patient monitoring.

By fostering interdisciplinary collaboration and responsible innovation, Mid-IR wearables may support early intervention, population-level insights, and more equitable healthcare delivery. In this way, they represent a potential pathway to reduce diagnostic delays, mitigate gender and access disparities, and support a shift toward preventive, patient-centered, and data-driven healthcare. Overall, Mid-IR wearable sensing highlights how emerging technologies, when coupled with robust validation and system-level integration, may contribute to a more anticipatory, patient-centered, and inclusive model of metabolic healthcare.

## Acknowledgments

The authors would like to thank Dr. Laura Avogaro for her revision and our medical writer Dr. Erika Bandini for her valuable support during the preparation of the manuscript. The authors also thank Dasha C. del Blanco for preparing the original illustrations used in Figures 1, 2, and 3. This work was supported by funding from the 10.13039/100018693European Union’s Horizon Europe program under the European Innovation Council Pathfinder Initiative, grant agreement ID 101115476.

## Author contributions

All authors (L.A., T.B., I.C., N.E., A.F., C.F., N.H., J.K., M.M., S.N., S.O.-P., T.P., H.S., R.S., M. Silvestrov, N.S., M. Straccia, O.S., H.V.L.-T., O.M.E.Y., and A.Z.) have conceptualized and reviewed the manuscript. I.C. and N.E. have also drafted and coordinated the manuscript drafting.

## Declaration of interests

Several authors are employed by private companies involved in the development, validation, or potential commercialization of wearable sensing, AI, and digital health technologies (AI Expert SRL, Quantune Technologies GmbH, and FRESCI by Science&Strategy SL). The remaining authors are affiliated with public research organizations (Joanneum Research Forschungsgesellschaft mbH, Radboud University Medical Center, and VTT Technical Research Center of Finland). The MiWear project is a collaborative research initiative, and the views expressed in this perspective reflect the authors’ scientific and professional opinions.
